# Supplementary figures and images for: Influence of Genotype on High Glucosinolate Synthesis Lines of Brassica rapa
Source: Int J Mol Sci. 2021 Jul 7;22(14):7301. doi: 10.3390/ijms22147301 (PMC8305852; doi:10.3390/ijms22147301)

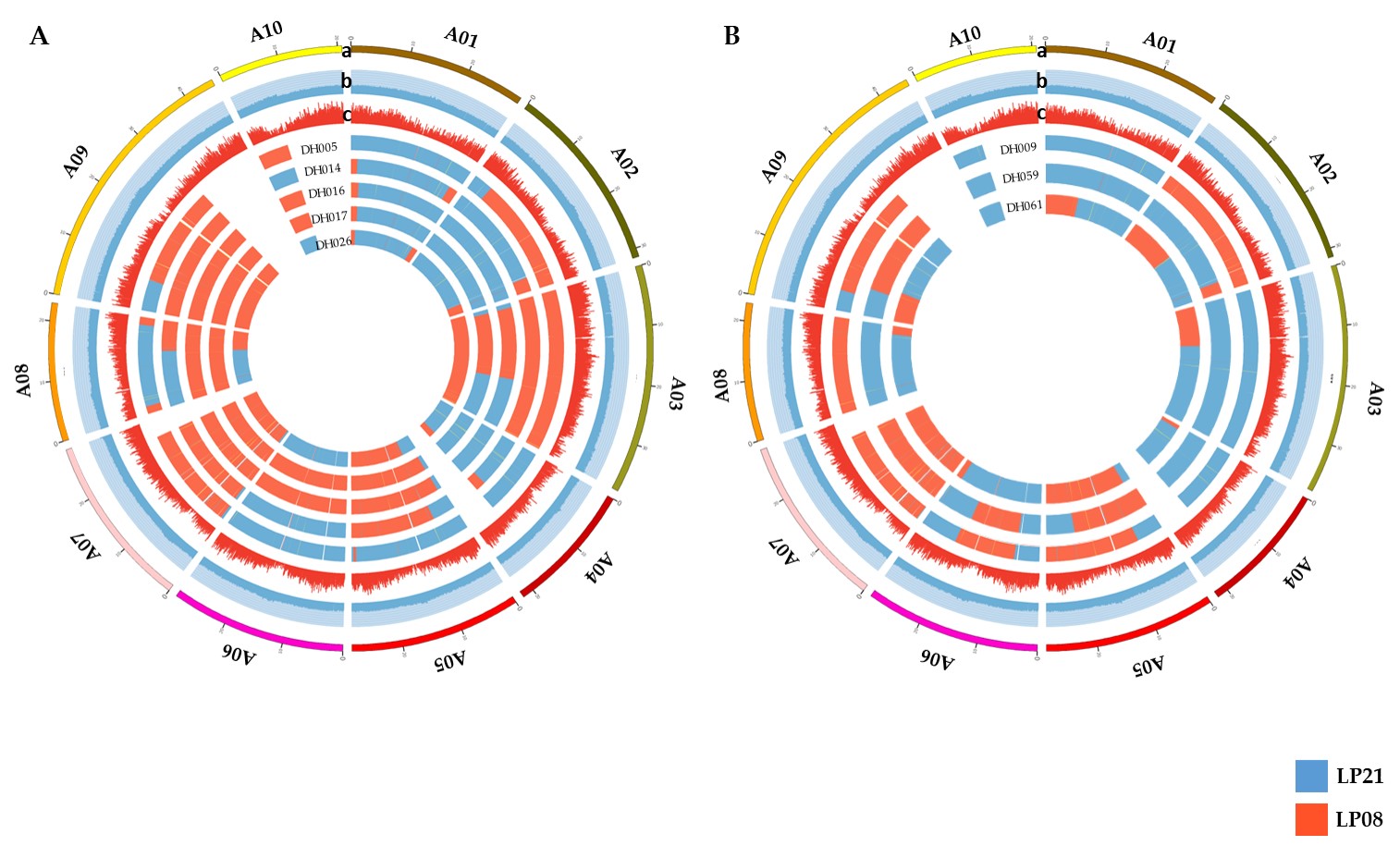

Supplement: Supplementary file 1 [file ijms-22-07301-s001.zip › Supplementary_Figure_1.jpg]
